# NLRX1 does not play a role in diabetes nor the development of diabetic nephropathy induced by multiple low doses of streptozotocin

**DOI:** 10.1371/journal.pone.0214437

**Published:** 2019-03-25

**Authors:** Angelique M. L. Scantlebery, Melissa Uil, Loes M. Butter, Renée Poelman, Nike Claessen, Stephen E. Girardin, Sandrine Florquin, Joris J. T. H. Roelofs, Jaklien C. Leemans

**Affiliations:** 1 Department of Pathology, Academic Medical Center, University of Amsterdam, Amsterdam, North Holland, The Netherlands; 2 Department of Laboratory Medicine and Pathobiology, University of Toronto, Toronto, Ontario, Canada; National Institutes of Health, UNITED STATES

## Abstract

Diabetic nephropathy (DN) is a microvascular complication of diabetes mellitus that results in both tubular and glomerular injury. Low-grade inflammation and oxidative stress are two mechanisms known to drive the progression of DN. Nucleotide-binding leucine-rich repeat containing family member X1 (NLRX1) is an innate immune receptor, uniquely located in mitochondria, that has been found to regulate inflammatory responses and to dampen renal oxidative stress by regulating oxidative phosphorylation. For this reason, we investigated the role of NLRX1 in the development of DN in a Type 1 Diabetes mouse model. We analyzed the effect of NLRX1 deficiency on diabetes development and the accompanied renal damage, inflammation, and fibrosis. We found that multiple low doses of streptozotocin induced body weight loss, polydipsia, hyperglycemia, glycosuria, and a mild DN phenotype in wildtype and NLRX1-deficient mice, without significant differences between these mouse strains. Despite increased NLRX1 expression in diabetic wildtype mice, NLRX1 deficiency did not affect the diabetic phenotype induced by streptozotocin treatment, as reflected by similar levels of polyuria, microalbuminuria, and increased renal markers of oxidative stress and inflammation in wildtype and NLRX1-deficient mice. The present findings show that NLRX1 does not mediate the development of streptozotocin-induced diabetes and diabetic-induced nephropathy in mice after multiple low doses of streptozotocin. This data implies that, while NLRX1 can be triggered by cellular stress, its regulatory and functional effects may be dependent on the specific physiological conditions. In the case of DN, NLRX1 may be neither helpful nor harmful, but rather a marker of metabolic stress.

## Introduction

Diabetic nephropathy (DN) remains the leading cause of end-stage renal disease (ESRD) in the developed world. It is clinically characterized by albuminuria, reduced glomerular filtration, and hypertension, with the latter leading to an increased risk for the development of cardiovascular disease[1]. Among many different pathogenic mechanisms, sustained low-grade inflammation and oxidative stress have been identified as pivotal key players in the development of DN[2–6].

In DN, proteinuria levels and renal function have been found to correlate with the degree of the inflammatory response[7,8]. Previous studies, on the inflammatory response associated with DN, have shown Toll-like receptors(TLRs), a family of Pattern Recognition Receptors(PRRs), to be involved in the perpetuation of renal inflammation during diabetes[9]. TLRs initiate pro-inflammatory signaling cascades in response to both pathogen- and damage-associated molecular patterns, known as PAMPs and DAMPs, respectively. Furthermore, TLR2 and TLR4 expression is associated with the level of macrophage infiltration, and their inhibition was shown to be reno-protective in DN models[10,11]. Oxidative stress is a physiological imbalance that occurs when antioxidant defense systems are incapable of keeping reactive oxygen species (ROS) within homeostatic levels. DN and oxidative stress are closely linked due to hyperglycemia-induced ROS production and the mitigation of antioxidant responses through the glycation of antioxidant enzymes. Several lines of evidence have established a causative relationship between oxidative stress and DN, and the inhibition of oxidative stress was found to reduce severity of the disease[12]. Excess ROS increases extracellular matrix (ECM) production, which progresses towards fibrosis and, eventually, ESRD[13].

Nucleotide-binding leucine-rich repeat containing family member X1 (NLRX1) is the most newly identified member of the NLR family. NLR proteins function within the innate immune system as PRRs. NLR proteins are grouped according their N-terminal domain. NLRX1 is unique in the fact that it contains an N-terminal domain that does not resemble that of the other NLR proteins, and it lacks the domains necessary for inflammasome formation. This same atypical N-terminal was found to enable NLRX1 to localize specifically to the mitochondrion. No other NLR protein is known to localize to a specific organelle[14,15]. The targeting of NLRX1 to the mitochondrion leads to ROS generation, a well-known microbial defense, and the activation of NF-κB and JNK signaling during the innate immune response[16]. Recently, our group showed that the effect of NLRX1 on mitochondrial ROS production may be different in the absence of a microbial trigger. In a model of renal ischemia reperfusion injury (IRI), we observed increased levels of oxidative stress in NLRX1-deficient mice, implying that NLRX1 could also reduce ROS production by regulating mitochondrial activity[17]. Indeed, NLRX1-deficient tubular epithelial cells have an enhanced oxidative phosphorylation status as compared to control cells[17].

Given the fact that NLRX1 has the unique ability to directly affect both innate immunity and oxidative stress, it is plausible that NLRX1 may play a role in DN. Based on the aforementioned evidences regarding the known effects of PRRs and NLRX1 on inflammation and oxidative stress, respectively, we have established 2 hypotheses in which NLRX1 could play either a protagonistic or antagonistic role in the pathogenesis of DN. Firstly, if NLRX1 functions in a manner similar to that of other PRRs, such as TLRs, we can hypothesize that NLRX1 would facilitate the development of DN through the enhancement of proinflammatory signaling. Secondly, if the inflammation in DN is considered to be sterile, as is the case in IRI, we can hypothesize that NLRX1 would protect against oxidative stress, thereby antagonizing the progression of DN.

We, therefore, assessed the role of NLRX1 in DN in a multiple low-dose streptozotocin (STZ) model of diabetes using wild type (WT) and NLRX1-deficient C57BL/6J male mice. Diabetic parameters were measured and renal tissue was examined for damage, inflammation, fibrosis, and oxidative stress.

## Materials and methods

### Mice

NLRX1^-/-^ mice were generated as previously described[18]. In short, floxed mice with loxP sites at Exon 3 were crossed with a C57BL/6J Cre mouse to generate a NLRX1-deficient C57BL/6J mouse lacking Exon 3. Male WT and NLRX1^-/-^ C57BL/6J mice (Charles River), 11±1 weeks old, weighing 27.5±2 grams, were used.

Animals were housed at the animal care facility of the Academic Medical Center (University of Amsterdam), in accordance with national guidelines, and given ad libitum access to both food and water. Mice were housed in enriched cage systems with 2–4 mice per cage. Twice a week, a health monitoring report was filled out by animal care staff which includes a scoring system for animal discomfort, overall health, responsiveness, physical movement, body weight stability, fur condition, and signs of fighting/aggression (especially among males). At the animal facility, all rooms operate as barriers with 100% fresh, HEPA-filtered air. The movement of persons, equipment, and samples follows pre-defined traffic patterns to prevent clean and dirty supplies from crossing paths. Environmental conditions such as temperature, relative humidty, air flow, light intensity, and management of light and dark periods were monitored daily. These were in accordance with the criteria specified by the American Association for Laboratory Animal Science (AALAS).

Group sizes were calculated based on a power analysis, possible unresponsiveness to STZ, and possible loss due to STZ toxicity. Mice were allocated to treatment or control groups by animal caretakers which ensured no *a priori* knowledge of group assignment and prevented subjective experimenter bias. All experiments were approved by the Animal Care and Use Committee of the University of Amsterdam.

### Experimental procedures

Male WT and NLRX1^-/-^ C57BL/J mice were each divided into diabetic (n = 14) and non-diabetic control (n = 8) groups, which resulted in a total of 4 experimental groups.

Diabetes was induced by means of intraperitoneal injections of 50mg/kg STZ (Sigma-Aldrich, Zwijndrecht, The Netherlands) in a maximum volume of 20ml/kg sodium-citrate buffer, for 5 consecutive days. WT and NLRX1^-/-^ non-diabetic controls received 5 intraperitoneal injections of 100μl sodium-citrate buffer. All injections were administered between 9 and 10 AM each day and in the same order. Body weight and blood glucose were monitored weekly for a period of 23 weeks. All measurements were taken in the same order, alternating between cages containing WT and NLRX1^-/-^ mice as well as non-diabetic and diabetic mice. In the final 3 weeks, food and water intake were monitored weekly. In the 24th week, mice were anesthetized (4% isofluorane/100% oxygen), and blood was collected via eye extraction followed by cervical dislocation. Blood was collected in microvette CB 300 lithium heparin capillary tubes (Sarstedt, Nümbrecht, Germany) and centrifuged for 10 minutes at 10,000rpm to separate the plasma fraction, which was stored at -80°C. Both kidneys were harvested and each divided into 2 equal transverse sections. One section was snap frozen in liquid nitrogen and the other fixed in formalin prior to paraffin embedding.

### Non-fasted measurements: Blood glucose and hemoglobin A1c

Weekly blood glucose measurements were performed using the Contour XT glucose meter (Ascensia diabetes Care, NJ, USA). Blood was sampled from the saphenous vein. The first drop of blood was wiped away and the second was used for the blood glucose measurement. Measurements were taken at the same time of day (between 11am and 1pm). The hemoglobin A1c (HbA1c) percentage was measured in whole blood and is used to determine the degree to which hemoglobin in the erythrocytes has become glycated. Whole blood was collected in the same manner as described for the blood glucose measurement, and HbA1c% was measured using the Mouse Hemoglobin A1c (HbA1c) Assay Kit (Crystal Chem, IL, USA, catalog# 80310).

### Fasted measurements: Insulin, blood glucose, and the Homeostatic model assessment of β-cell function (HOMA- β)

Following a 4-hour fast, fasting blood insulin and glucose were measured. Blood glucose levels were measured as mentioned previously, and insulin levels were measured in plasma using the Ultra-Sensitive Mouse Insulin ELISA kit (Crystal Chem, IL, USA, catalog #90080).

The HOMA-β percentage was calculated as follows:

[20 * fasting insulin (mU/l)] / [fasting blood glucose (mmol/l)– 3.5][19].

### Urine analyses: Urinary output, Urinary Albumin Excretion (UAE), and urinary glucose

Mice were housed in metabolic cages for 24 hours in order to obtain 24-hour urine for the measurement of Urinary Albumin Excretion (UAE). The urine volume collected per mouse was documented as urinary output in grams. UAE was measured using the Mouse albumin ELISA quantification kit (Bethyl Laboratories, TX, USA, cat no E90-134, ITK Diagnostics). UAE was corrected for urinary output per mouse. Urinary glucose was determined by a spectrophotometric measurement of glucohexokinase activity. This automated measurement was performed in the Roche cobas c702 Chemistry Analyzer at the Laboratory for General Clinical Chemistry (LAKC) at the Academic Medical Center of Amsterdam.

### Plasma analyses: Creatinine and urea

Plasma creatinine was measured by means of a colorimetric enzyme assay, and plasma urea was determined by means of a kinetic-spectrophotometric quantification of urease activity. Both measurements were performed in an automated system (Roche cobas c702 Chemistry Analyzer) at the Laboratory for General Clinical Chemistry (LAKC) at the Academic Medical Center of Amsterdam.

### Histochemical stainings

Kidney sections underwent PAS-D staining to allow for general histological analysis. Formalin-fixed paraffin embedded (FFPE) tissue was deparaffinized and rehydrated. Tissue sections were incubated in a 0.25% amylase solution (Type VI-B, Sigma Aldrich, Missouri, USA), followed by an incubation in 1% periodic acid (Merck), and were finally incubated in Schiff’s reagent (Merck, Darmstadt, Germany). Hematoxylin solution (50%, Klinipath, Duiven, The Netherlands) was used as a nuclear stain, after which sections were dehydrated and sealed with Pertex.

### Immunohistochemical stainings

Macrophages (F4/80), lipid peroxidation [4-hydroxynonenal (4-HNE)], collagen I and collagen IV were detected in kidney sections by means of immunohistochemical stainings. FFPE tissue was deparaffinized and rehydrated. Sections were incubated in 0.3% H_2_O_2_ in methanol to block endogenous peroxidase and boiled in 0.01M pH 6.0 citrate buffer for epitope retrieval. For macrophage detection, kidney sections were incubated with *rat IgG2b-anti-F4/80* (Bio-connect, mca497ga) primary antibody followed by *rabbi-anti-rat* (Dako) secondary antibody.

For the detection of lipid peroxidation products, kidney sections were incubated with *anti-mouse 4-HNE* mAb primary antibody (Abcam, Ab46545).

For the detection of collagen I and collagen IV, kidney sections were incubated with *rabbit anti- collagen I* (GeneTex, gtx41286) and *rabbit anti-collagen IV* pAb primary antibodies (Abcam, Ab6586), respectively.

F4/80-, 4-HNE-, collagen I, and collagen IV-stained sections were incubated with a tertiary, peroxidase-labeled Powervision poly HRP-anti rabbit antibody (Immunologic, Duiven, The Netherlands) prior to visualization.

F4/80- and collagen I-stained sections were visualized with 1% H_2_O_2_ and 3,3'-Diaminobenzidine (DAB) (Sigma-Aldrich) in 0.05M Tris-HCL, while 4-HNE- and collagen IV-stained sections were visualized with the DAB Plus system (Dako).

Hematoxylin solution (50%, Klinipath, Duiven, The Netherlands) was used as a standard nuclear stain, after which sections were dehydrated and sealed with Pertex. All primary antibody incubations were performed overnight at 4°C. All secondary and tertiary antibody incubations were performed for 30 minutes at RT.

### Quantification of (immuno)histochemical stainings

Mesangial cellularity was manually quantified in PAS-D-stained renal sections. Fifty glomeruli were counted per mouse. Mesangial hypercellularity was confirmed per glomerulus when more than 3 mesangial cells were observed in a row. For the F4/80, 4-HNE, and collagen I stainings, 10 high-power-field (HPF) (40x magnification) images were captured of the renal cortex and the percentage of the positively stained area was digitally quantified using ImageJ software. Cortical and glomerular collagen IV deposition were digitally quantified in 15 HPFs (40x magnification) of the renal cortex and 15 glomeruli, respectively, using Image J software.

### Real time-qPCR

Total RNA was isolated from 300um-thick frozen renal sections using TRIzol (Invitrogen, Breda, The Netherlands) reagents. RNA isolation was performed according to the manufacturer’s instructions. Complementary DNA was generated following a standard protocol. Briefly, oligo-dT primers were ligated to the RNA for 10 minutes at 72°C, followed by a 60 minute polymerization at 37°C with M-MLV reverse transcriptase (Thermo Fisher Scientific). Real time cDNA quantification was performed on the Roche LightCycler 480 (Roche Diagnostics, Woerden, The Netherlands) using SensiFAST SYBR NO ROX kit (Bioline, London, UK). Gene expression was normalized against *TATA sequence binding protein* (TBP) expression. Analysis was performed using the LinRegPCR 12.4 software[20]. The following sequences (synthesized by Eurogenetec, Maastricht, The Netherlands) were used for the analysis of NLRX1: (forward: TGCCATTTGCCCAGGACCTCTT, reverse: GCTCCACTGGATCAAGAAGGAGATATGC) and TBP: (forward GGAGAATCATGGACCAGAACA:, reverse: GATGGGAATTCCAGGAGTCA).

### Western blot

Snap frozen kidney sections were homogenized in Greenberger Lysis buffer (150mM NaCl, 15mM TRIS, 1mM MgCl.6H_2_O, 1mM, CaCl2.2H_2_O, 1% Triton) and set to pH 4 with HCL, containing a protease inhibitor cocktail (Sigma Aldrich). Samples were lysed for 30 minutes on ice, after which the samples were spun down for 10 minutes at 4°C. The supernatants were stored in new Eppendorf tubes and used for western blot analysis. Protein concentration was determined by means of the Bicinchoninic Acid Assay (BCA) (Thermo Fisher Scientific, Waltham, MA, USA) to allow for the preparation of 10μg protein in sample buffer, containing 2-mercapto-ethanol. Samples were subjected to gel electrophoresis for approximately 30 minutes at 200V. Semi-dry transfer was performed using the Thermo Scientific Pierce Power System. Membranes were blocked in 5% milk in Tris-buffered saline (TBS), containing 0.5% Tween-20 for 1 hour. This was followed by overnight incubation in *mouse-anti-mouse α-SMA IgG2A* primary antibody (1:1000, clone 1A4, M085101, DAKO) at 4°C and a 2-hour incubation with secondary antibody *goat-anti-mouse IgG2A-HRP* (1:5000, Southern Biotech) at RT. Beta-actin was measured as a loading control using the primary antibody, *mouse-anti-mouse β-actin IgG1* (1:5000, cat SA5316, Sigma-Aldrich), and the secondary antibody, *goat-anti-mouse IgG1-HRP* (1:5000, Thermofisher Scientific). Membranes were treated with ECL reagents (Thermofisher Scientific) and visualized using the LAS 4000 system. Results were digitally quantified using Image J software and normalized to the loading control.

### ELISA

Kidney homogenates were used to measure protein expression of Monocyte chemoattractant protein 1 (MCP-1), Interleukine-6 (IL-6), Tumor necrosis factor-alpha (TNF-α), and active Transforming Growth Factor Beta 1 (TGF-β1) using ELISA duosets (R&D Systems, Abingdon, UK). ELISAs were performed according to the manufacturer’s instructions and corrected for protein concentration as determined by the BCA assay (Thermo Fisher Scientific, Waltham, MA, USA).

### Statistical analysis

Data were anlayzed using Graphpad Prism 5 software and are shown as mean ± SEM. Data was tested for normality using the D’Agostino & Pearson omnibus normality tests. Normally distributed data was statistically analyzed using One-way ANOVA with Bonferroni correction. Data sets with a non-normal distribution were statistically analyzed using the Kruskal Wallis test with Dunn’s correction. The Grubbs test was used to identify outliers, which were then excluded from the analysis. As the HbA1c score reflects glycemic levels over a 3-month period, it was considered to be one of the most reliable criterion for the identification of non-responders. Furthermore, a positive correlation was found between HbA1c levels and fasting glucose measurements (R = 0.84, S1 Fig). Mice with a HbA1c measurement below 5% were considered non-responders and were excluded from all analyses. This led to the exclusion of 2 mice in the WT diabetic group and 5 mice in the NLRX1-deficient diabetic group.

## Results

### NLRX1 does not contribute to the development of diabetes in mice after multiple low doses of STZ

**Fig 1:**
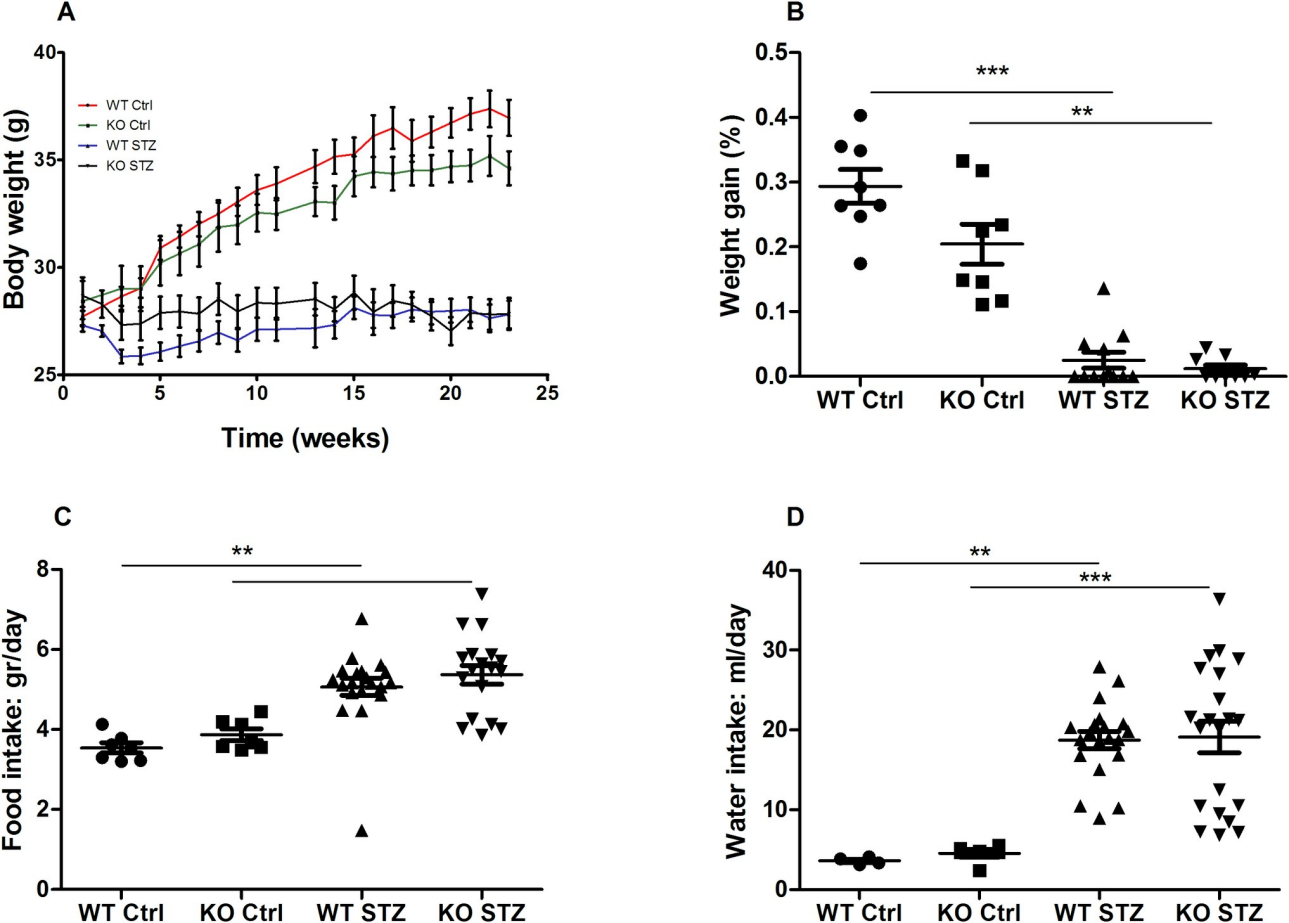
Metabolic parameters: Body weight, weight gain, food intake, and water intake. (A) Body weight measured weekly for 24 weeks. (B) Weight gain calculated as a percentage of original body weight. (C-D) Food and water intake measured in the final 3 weeks of the study. Data shown as mean ± SEM. **p<0.01, ***p<0.001.

To determine the efficacy of the model and confirm the induction of diabetes, several metabolic and clinical parameters were tested. Body weight and weight gain were measured in all groups. A steady increase in weight gain was observed in non-diabetic controls, but this was impaired in both diabetic groups (Fig 1A). Weight gain, when calculated as a percentage of the original body weight, was found to be significantly decreased in diabetic mice with no significant differences between the two strains. In non-diabetic controls, a trend towards decreased weight gain was observed in NLRX1-deficient mice compared to WTs (Fig 1B). A significant increase in both food and water intake (polydipsia) was observed in diabetic mice compared to non-diabetic controls, without differences being observed between the mouse strains (Fig 1C and 1D).

**Fig 2:**
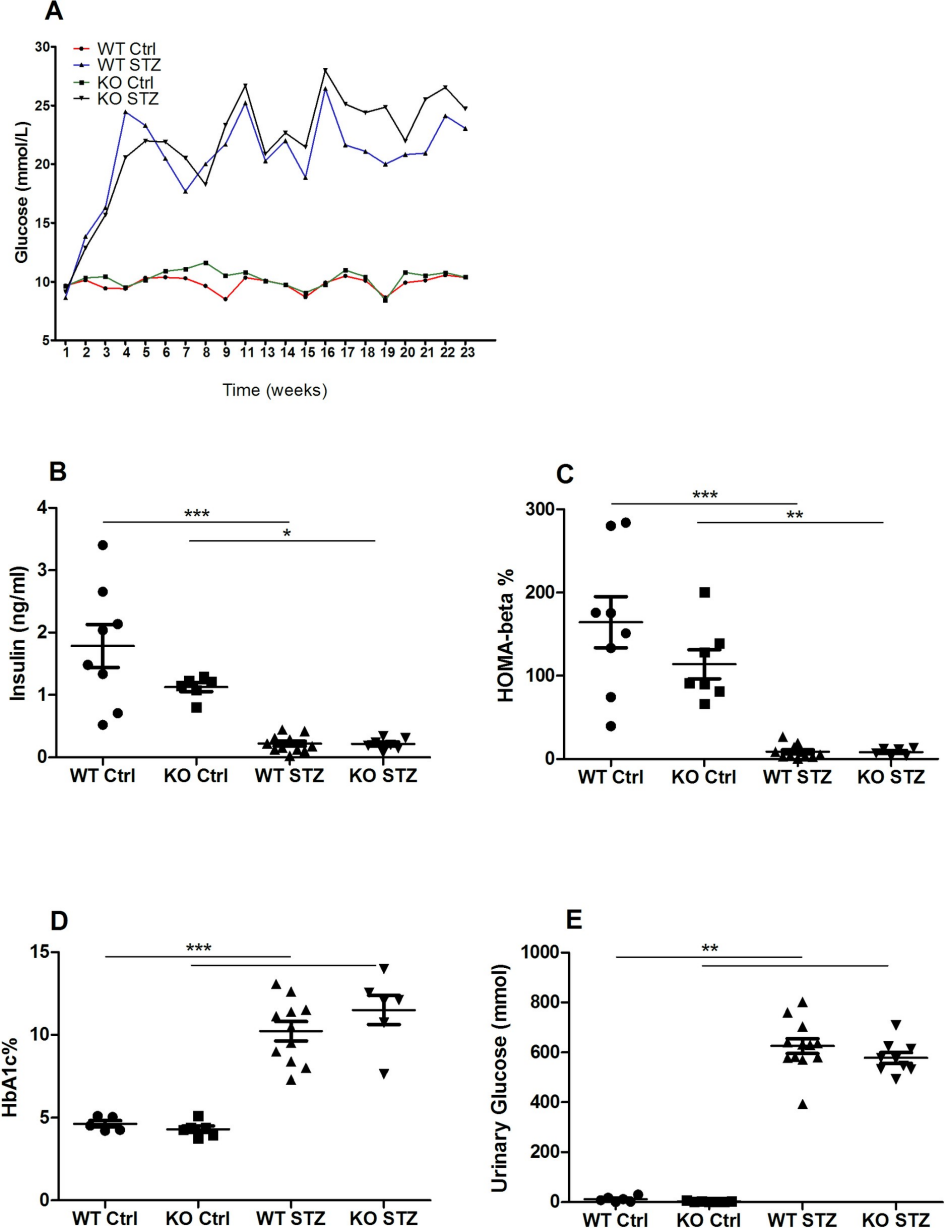
Diabetic parameters: Blood glucose, fasting insulin, HOMA-beta, HbA1c, and urinary glucose. (A) Weekly blood glucose measured with the Contour XT glucose meter. (B) Fasting plasma insulin measured with the Ultra-Sensitive Mouse Insulin ELISA kit. (C) HOMA-beta percentage was calculated using fasting blood glucose and plasma insulin values. (D) HbA1c measurement was measured in whole blood using the Mouse Hemoglobin A1c (HbA1c) Assay Kit. (E) Urinary glucose excretion measured in 24-hour urine in the automated Roche cobas c702 Chemistry Analyzer. Data shown as mean ± SEM. *p<0.05, **p<0.01, ***p<0.001. HOMA-beta = Homeostatic model assessment of β-cell function; HbA1c = Hemoglobin A1c.

All mice were monitored with weekly blood glucose measurements, and 12 weeks following STZ treatment, HbA1c% was measured to determine the level of glycated hemoglobin. As STZ induces diabetes by the depletion of insulin-secreting pancreatic beta cells, fasting insulin levels and the functionality of the beta cells (HOMA-beta) were determined for all mice. Blood glucose levels were elevated and unstable in diabetic mice when compared to non-diabetic controls, whose blood glucose levels remained relatively unchanged throughout the study (Fig 2A). Both fasting insulin and HOMA-beta levels were significantly decreased in both diabetic groups. In non-diabetic controls these parameters were lower, but not significantly (n.s.), in NLRX1-deficient mice compared to WTs (Fig 2B and 2C). STZ treatment resulted in a significant increase in HbA1c% in both diabetic groups (Fig 2D). Urinary glucose excretion (glycosuria) was significantly increased in diabetic mice (Fig 2E). For all parameters, no significant differences were observed between WT and NLRX1-deficient mice.

### NLRX1 does not contribute to the development of polyuria and microalbuminuria in STZ-induced diabetic mice

**Fig 3:**
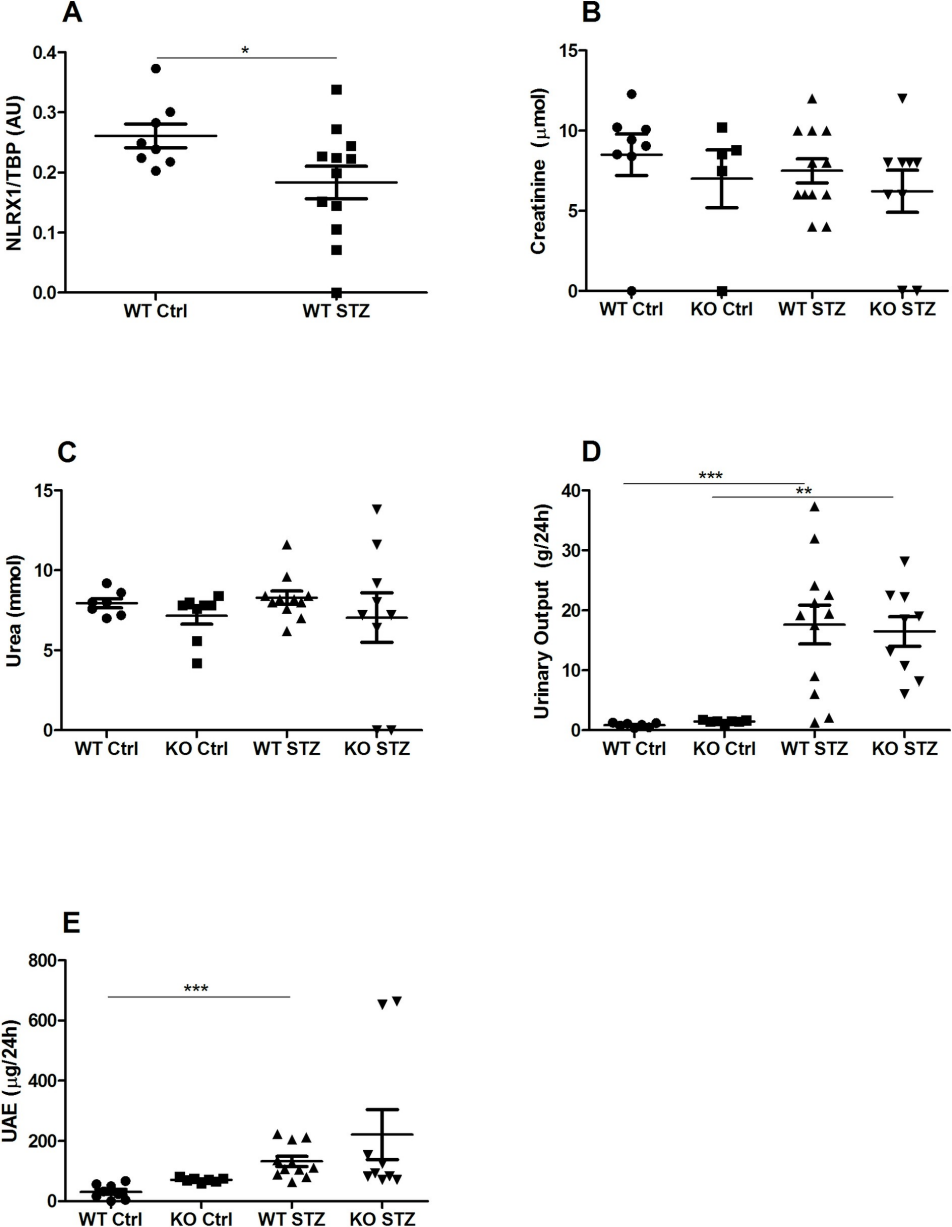
Functional renal markers: NLRX1 mRNA expression, plasma creatinine, plasma urea, urinary output, and urinary albumin excretion. (A) NLRX1 mRNA expression measured in renal tissue of non-diabetic and diabetic WT mice. (B-C) Plasma creatinine and plasma urea measured in the automated Roche cobas c702 Chemistry Analyzer. Urinary output and UAE measured in 24-hour urine. (D-E) UAE was measured using the Mouse albumin ELISA quantification kit. Data shown as mean ± SEM.*p<0.05, **p<0.01, ***p<0.001. UAE = Urinary albumin excretion.

Several analyses were performed to assess the effects of NLRX1 deficiency and STZ treatment on renal function. Furthermore, the effect of diabetes on NLRX1 expression was examined in WT mice. STZ treatment resulted in a significant decrease in renal NLRX1 mRNA expression (Fig 3A). Plasma creatinine and urea levels were comparable between non-diabetic controls and diabetic mice, indicating the absence of overt renal dysfunction (Fig 3B and 3C). Urinary output, however, was significantly increased in diabetic mice, compared to non-diabetic controls, with no difference being observed between WT and NLRX1-deficient mice (Fig 3D). Urinary albumin excretion was increased in both diabetic groups, but statistical significance was only achieved in WT diabetic mice (Fig 3E).

### NLRX1 does not contribute to the development of renal oxidative stress and inflammation in STZ-induced diabetic mice

**Fig 4:**
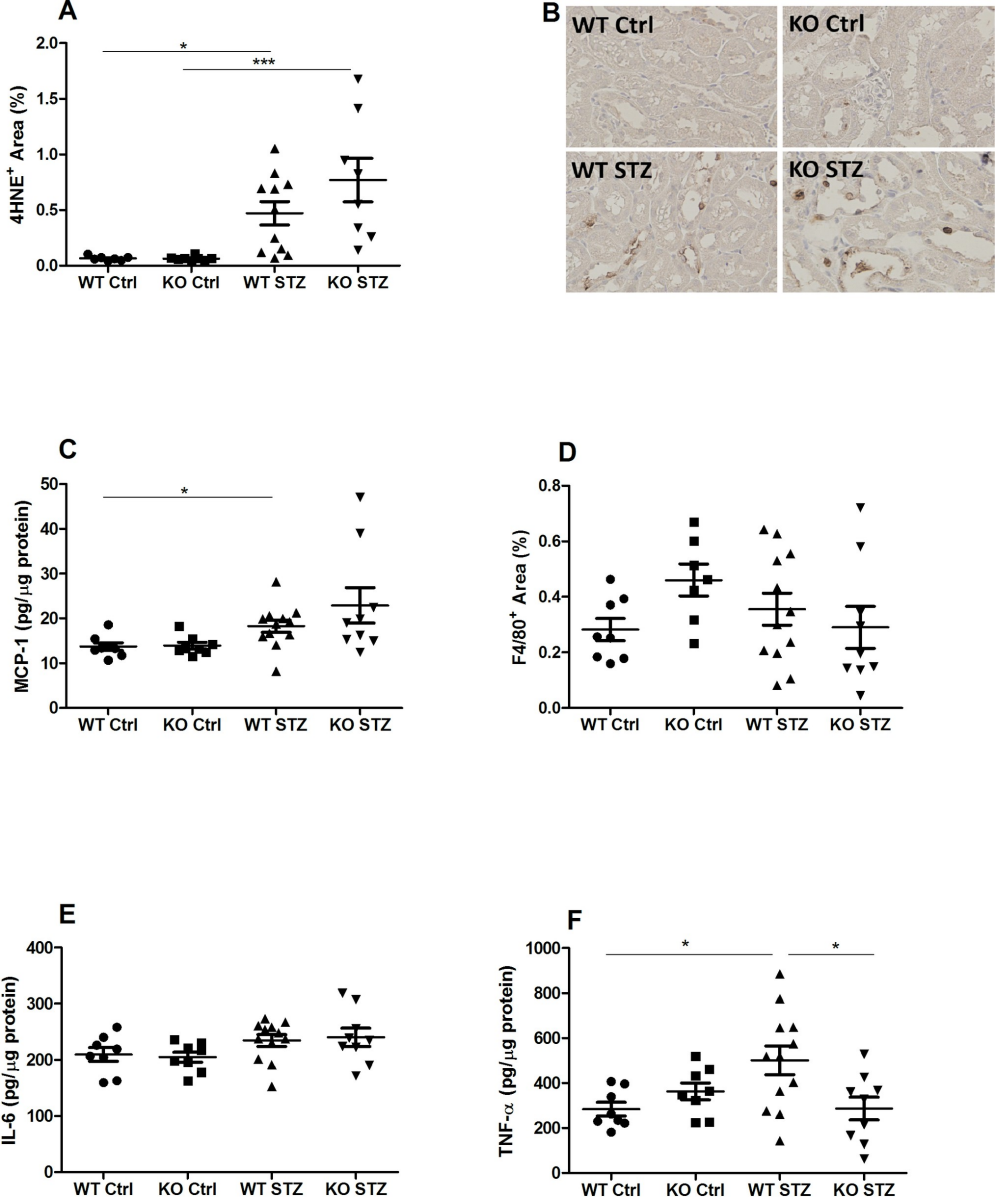
Oxidative stress and inflammation: 4-HNE, F4/80, MCP-1, IL-6, and TNF-α. (A) Quantification of the 4HNE staining in renal tissue. (B) Representative photos of the 4HNE staining (40x magnification). (C) MCP-1 protein expression in kidney homogenate measured by ELISA. (D) Quantification of F4/80 staining for cortical renal macrophages. (E-F) Expression of pro-inflammatory cytokines IL-6 and TNF-α measured by ELISA in kidney homogenate. Data shown as mean ± SEM. *p<0.05, **p<0.01, ***p<0.001. 4HNE = 4-hydroxynonenal; MCP-1 = Monocyte chemoattractant protein 1; F4/80 = macrophage marker; IL-6 = Interleukin-6; TNF-α = Tumor necrosis factor alpha.

As hallmarks of DN, both oxidative stress and inflammatory markers were analyzed in renal tissue. The presence of the lipid peroxidation product, 4-HNE, a marker for oxidative stress, was significantly increased in both WT and NLRX1-deficient, STZ-treated mice (Fig 4A and 4B). Renal inflammation was measured by quantifying MCP-1 expression, the presence of macrophages (F4/80), and macrophage-derived cytokines,IL-6 and TNF-α. MCP-1 was increased in both diabetic groups when compared to controls, but statistical significance was only achieved in WT diabetic mice (Fig 4C). The F4/80 staining was used for the identification of macrophages infiltrating the renal cortical interstitium. There was no difference in the number of F4/80-positive cells observed in non-diabetic controls and diabetic mice (Fig 4D). Similarly, IL-6 levels were not affected by the diabetic condition in either mouse strain (Fig 4E). TNF-α was significantly increased in diabetic WTs, but remained unchanged in diabetic NLRX1-deficient mice, when compared to non-diabetic controls. Consequently, TNF-α was significantly lower in diabetic NLRX1-deficient mice compared to diabetic WT mice (Fig 4F).

### Multiple low doses of STZ led to a mild DN phenotype in both WT and NLRX1-deficient mice

**Fig 5:**
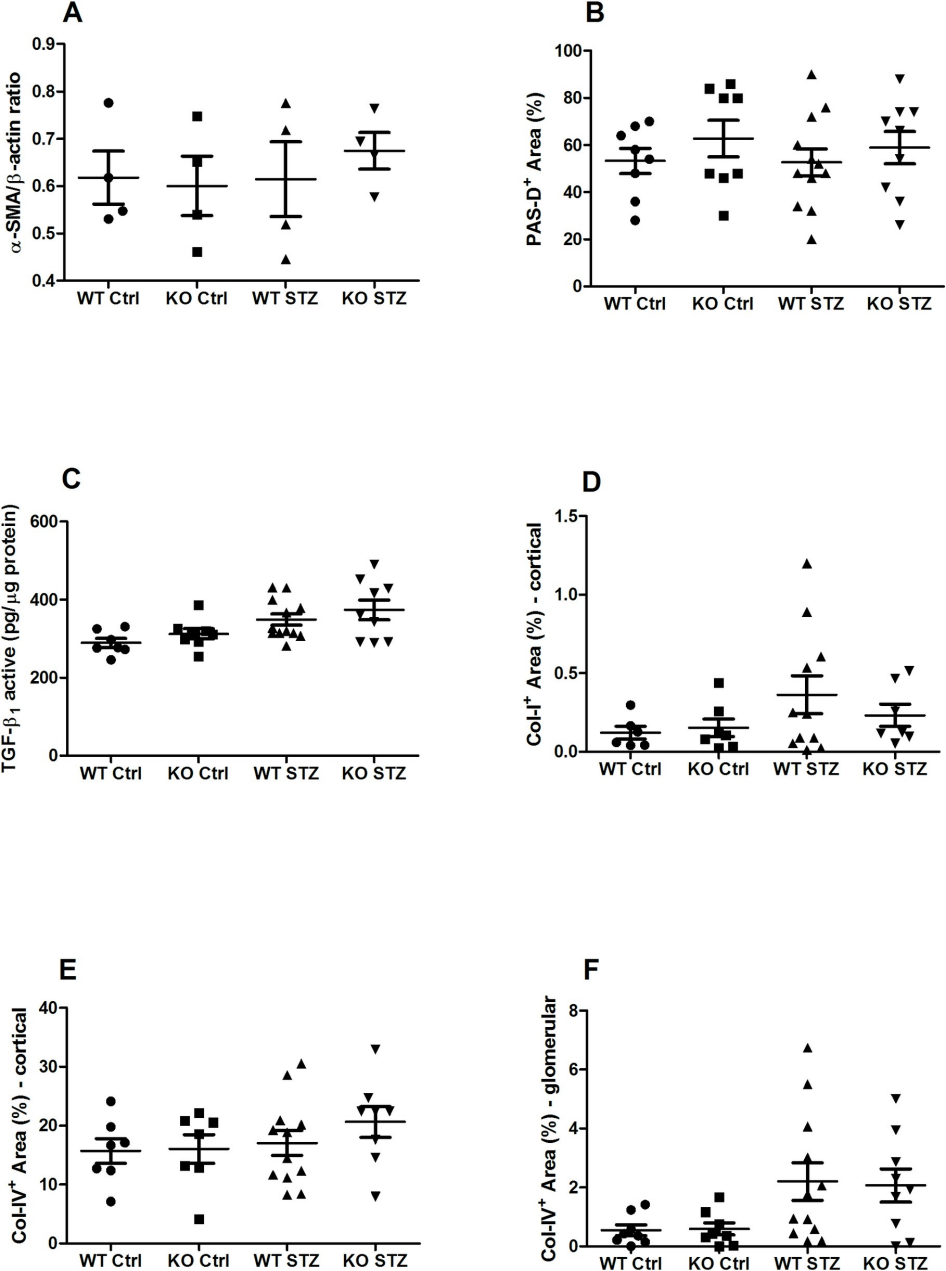
Renal fibrosis markers:, α-SMA, mesangial cellularity active TGF-β1, collagen I, and collagen IV. (A) Expression of α-SMA mRNA in renal tissue. (B) Quantification of mesangial cellularity in PAS-D-stained renal sections. (C) Protein expression of active TGF-β1, measured by ELISA in kidney homogenate. (D-F) Quantification of collagen I (cortical) and collagen IV (cortical and glomerular) stainings in renal tissue. Data shown as mean ± SEM. α-SMA = alpha smooth muscle actin; TGF-β1 = Transforming Growth Factor-Beta 1.

Structural renal alterations and fibrosis were analyzed by the histological assessment of the glomerular mesangium and the presence of known fibrotic markers. The diabetic state did not affect renal α-SMA accumulation, mesangial cellularity, or active TGF-β1 expression in WT and NLRX1-deficient mice, indicating a mild DN phenotype (Fig 5A–5C). A trend towards increased collagen 1 deposition was observed in WT, but not in NLRX1-deficient, diabetic mice (Fig 5D). A trend towards increased cortical collagen IV deposition was observed in NLRX1-deficient, but not WT diabetic mice when compared to non-diabetic controls (Fig 5E). A trend towards increased glomerular collagen IV expression was observed in both diabetic groups with no differences observed between the mouse strains (Fig 5F).

## Discussion

DN, a renal complication of diabetes, is the leading cause of ESRD worldwide. Its pathogenesis is comprised of several features, including low-grade inflammation and oxidative stress. NLRX1 is the most newly discovered member of a family of PRRs that affects inflammation, but also ROS production, due to its localization in the mitochondrion. Reports have indicated that other PRRs, namely TLR2/4, are involved in the progression of DN and that their inhibition improved disease outcome [21]. This gave further plausibility to our hypothesis that NLRX1 may affect the pathogenesis of DN. This study aimed to determine whether NLRX1 played a role in the development of diabetes and DN after multiple low-dose STZ treatment. Our results show that the lack of NLRX1 does not affect the development nor severity of diabetes and DN.

The multiple low-dose STZ model was successful in the initiation of Type-1 diabetes mellitus (T1DM) in both WT and NLRX1-deficient mice. A diabetic phenotype was clearly established, as seen in increased blood glucose, HbA1c values, polyuria, glycosuria, oxidative stress, and inflammation. Interestingly, the effects of an NLRX1 deficiency can be observed at basal levels. A trend towards less weight gain and lower insulin levels, the latter of which also resulted in lower HOMA-beta scores, is observed in NLRX1-deficient non-diabetic controls. However, disease severity did not differ between WT and NLRX1-deficient mice, as demonstrated by the similar results observed upon the assessment of diabetic parameters in both diabetic groups.

The hyperglycemia induced in this model gave rise to a mild DN phenotype, which is seen most clearly by the increase in UAE and oxidative stress (4HNE). However, these parameters were not affected by the NLRX1 deficiency. Renal damage and dysfunction could not be clearly measured in diabetic mice, despite the presence of increased UAE. Fibrosis could not be detected in α-SMA expression, mesangial hypercellularity, nor cortical collagen I and IV accumulation. The trends towards increased glomerular collagen IV expression the only sign of (early) fibrosis observed in the diabetic mice. Based on the data gathered in this study, we can infer that NLRX1 does not play a role in this mild DN phenotype, however the effect of NLRX1 in a more severe DN model requires further investigation. Furthermore, we are aware of the fact that C57BL/6J mice are relatively resistant to nephropathy[22]. So, while it may be difficult to assess nephropathy in this model, clear conclusions can be made upon the severity of the diabetic condition, as these mice are highly responsive to STZ treatment. An *in vivo* model more susceptible to nephropathy may be useful in determining whether reduced NLRX1 expression in DN elicits a functional effect[23]. We do acknowledge the significant decrease in NLRX1 mRNA expression observed in WT mice upon diabetes but are hesitant to presume that this may indicate a, yet unknown, role for NLRX1 in diabetes or DN. Alternatively, this may be a common response to renal damage or stress, as NLRX1 expression was also found to be decreased in human kidneys following ischemic injury and acute cellular rejection[17].

Due to its increased prevalence, one-third of all ESRD patients are type 2 diabetics. DN has a complex pathogenesis, which is further compounded by age in type 2 diabetics. As Type-2 diabetes mellitus (T2DM) is usually diagnosed around the age of 40, type 2 diabetics that suffer from nephropathy may show exacerbated features of the disease due to the higher prevalence of hypertension, obesity, and/or smoking in this age group[24]. We, therefore, used a T1DM model due to the reduced number of confounders that would have to be taken into consideration when attempting to recapitulate the human situation. Interestingly, our group has recently shown NLRX1-deficient, western-diet-fed, mice to be protected against renal fat accumulation and dysfunction[25]. A role for NLRX1 in diabetes and DN may, in fact, be better explored in a T2DM model, as type 2 diabetes is often associated with obesity. This hypothesis was recently tested by Costford *et al*., using a diet-induced T2DM model in WT and NLRX1-deficient C57BL/6N mice. They reported that NLRX1-deficient mice were partially protected from the development of the diet-induced hyperglycemia based on fasting glucose and insulin levels, as well as glucose tolerance. Interestingly, there were no genotype differences in body weight or adiposity. Histological investigation showed NLRX1-deficient mice to be protected from pancreatic lipid accumulation. Kidney hypertrophy was not observed in this study and renal damage was not reported, therefore, further investigation would be required to determine whether this partial protection from hyperglycemia would also afford protection against the development of DN in NLRX1-deficient mice[26].

In our study, the systemic, low-grade inflammation that is commonly associated with DN, was not clearly observed. STZ treatment was not found to affect the number of macrophages detected in renal sections from either mouse strain, despite the increased expression of MCP-1 in diabetic WT mice. TNF-α, which unlike IL-6, is primarily produced by macrophages, was found to be significantly increased in WT diabetic mice, but remained relatively unchanged in NLRX1-deficient diabetic mice. The cause for this significant difference in TNF-α expression observed between both diabetic groups is unclear as other markers (MCP-1 and F4/80) do not indicate differences in renal inflammation levels between WT and NLRX1-deficient diabetic mice.

In the context of T1DM, studies in non-obese diabetic (NOD) mice have shown TNF-α to be involved in disease onset, specifically through its effect on the autoimmune response towards pancreatic beta cells[27,28]. These findings are in line with the prominent role TNF-α is known to play in autoimmunity[29]. Therefore, in a classical, autoimmune-driven, T1DM model, decreased TNF-α levels would be expected have a marked effect. In the STZ model, where autoimmunity is not involved, we would not expect TNF-α to have a marked effect on disease onset. In this study, TNF-α, as well as IL-6, levels were measured as indicators of macrophage presence and activation for the assessment of the inflammatory response in the diabetic kidney, which is known to promote DN. Although we cannot explain the difference in the effect of the diabetic condition on TNF-α expression in the mouse strains, it remains that disease progression and development were found to be similar in both WT and NLRX1-deficient mice.

Based on our previous study[17], in which NLRX1-deficient mice were shown to be more susceptible to oxidative stress. We were, therefore, surprised to observe quite similar levels of lipid peroxidation in both WT and NLRX1-deficient diabetic mice. The oxidative stress model used in this previous study was achieved by inducing renal IRI. In this model, the effect of NLRX1 on oxidative stress was only noticeable after ischemia, which is accompanied by a strong influx of inflammatory cells and necrotic debris[17]. Although oxidative stress was also achieved in our current diabetic model, based on 4-HNE positivity, the inflammatory component and cellular injury was lacking. This may have abated the effect of NLRX1 on oxidative stress in our diabetic model.

In the previously described oxidative stress model, *in vitro* analysis of murine proximal tubular epithelial cells (PTECs), showed that NLRX1 deficiency affected cellular metabolism. NLRX1-deficient PTECs showed enhanced oxidative phosphorylation and a slight metabolic shift away from glycolysis[17]. Although glycolysis is far less efficient in ATP production, it metabolizes glucose much faster than oxidative phosphorylation. Interestingly, this NLRX1-dependent metabolic shift could have paradoxical effects in a diabetic setting.

We can theoretically speculate on the effects of a preference for glycolysis in WT PTECs and oxidative phosphorylation in NLRX1-deficient PTECs in the context of a diabetic model. Firstly, due to their higher rates of glycolysis, WT diabetic mice may be better able to metabolize the excess glucose in their system. Secondly, OXPHOS leads to more ROS production, and, therefore, more oxidative stress. During IRI, NLRX1 protected against renal oxidative stress, presumably by reducing tubular OXPHOS, which resulted in less ROS production and associated collateral renal damage[17]. In the context of DN, less oxidative stress may result in a milder phenotype. Based on this view, the NLRX1-driven propensity to metabolize glucose through glycolysis rather than OXPHOS would protect against diabetes and DN by increasing glucose expenditure and reducing oxidative stress levels.

On the contrary, OXPHOS has been linked to a more anti-inflammatory phenotype in macrophages[30,31]. As low-grade inflammation is not only a hallmark, but a key player in the progression of DN, inhibition of the immune response could be beneficial in delaying its development. In this way, the presence of NLRX1 could lead to a more inflammatory phenotype, through its partiality for glycolysis, which would expedite the progression of DN.

The modulatory effects of NLRX1 on ROS production and inflammation may be dependent on the physiological conditions. This is clearly exemplified in the NLRX1-dependent increase in ROS production observed during infection versus the NLRX1-dependent decrease in ROS production under ischemic conditions[16,32]. These seemingly inconsistent results may, in fact, be a reflection of the sensitivity of this receptor to changes in the cellular environment, thereby making it a key player in the maintenance of both metabolic and immunologic homeostasis.

## Conclusion

Based on the evidence gathered in this study, we conclude that NLRX1 does not mediate diabetes and diabetes-induced nephropathy in mice after multiple low doses of STZ. We believe that this may further demonstrate how NLRX1 differs from other NLRs and PRRs that do play a role in diabetes and DN.

## Supporting information

S1 Fig Correlation HbA1c% and fasting glucose levels in diabetic and normo glycemic mice. A positive correlation (R = 0.84, p<0.0001) was found between HbA1c% and fasting glucose values.
(TIF)

## References

[1] Najafian B, Mauer M. Progression of diabetic nephropathy in type 1 diabetic patients. Diabetes Res Clin Pract. 2009;83: 1–8. 10.1016/j.diabres.2008.08.024 19070384

[2] Saraheimo M, Teppo A-M, Forsblom C, Fagerudd J, Groop P-H. Diabetic nephropathy is associated with low-grade inflammation in Type 1 diabetic patients. Diabetologia. 2003;46: 1402–1407. 10.1007/s00125-003-1194-5 12928771

[3] Arora MK, Singh UK. Oxidative stress: meeting multiple targets in pathogenesis of diabetic nephropathy. Curr Drug Targets. 2014;15: 531–8. Available: http://www.ncbi.nlm.nih.gov/pubmed/24655140 24655140 10.2174/1389450115666140321120635

[4] Shen GX. Mitochondrial dysfunction, oxidative stress and diabetic cardiovascular disorders. Cardiovasc Hematol Disord Drug Targets. 2012;12: 106–12. Available: http://www.ncbi.nlm.nih.gov/pubmed/23030449 23030449 10.2174/1871529x11202020106

[5] Toyama T, Shimizu M, Furuichi K, Kaneko S, Wada T. Treatment and impact of dyslipidemia in diabetic nephropathy. Clin Exp Nephrol. 2014;18: 201–5. 10.1007/s10157-013-0898-1 24198049

[6] Yamahara K, Yasuda M, Kume S, Koya D, Maegawa H, Uzu T. The role of autophagy in the pathogenesis of diabetic nephropathy. J Diabetes Res. 2013;2013: 193757 10.1155/2013/193757 24455746 PMC3877624

[7] Kelly KJ, Dominguez JH. Rapid progression of diabetic nephropathy is linked to inflammation and episodes of acute renal failure. Am J Nephrol. 2010;32: 469–75. 10.1159/000320749 20956853

[8] Mezzano S, Aros C, Droguett A, Burgos ME, Ardiles L, Flores C, et al NF-kappaB activation and overexpression of regulated genes in human diabetic nephropathy. Nephrol Dial Transplant. 2004;19: 2505–12. 10.1093/ndt/gfh207 15280531

[9] Leemans JC, Kors L, Anders H-J, Florquin S. Pattern recognition receptors and the inflammasome in kidney disease. Nat Rev Nephrol. 2014;10: 398–414. 10.1038/nrneph.2014.91 24890433

[10] Lin M, Yiu WH, Wu HJ, Chan LYY, Leung JCK, Au WS, et al Toll-like receptor 4 promotes tubular inflammation in diabetic nephropathy. J Am Soc Nephrol. 2012;23: 86–102. 10.1681/ASN.2010111210 22021706 PMC3269929

[11] Maharjan AS, Pilling D, Gomer RH. Toll-like receptor 2 agonists inhibit human fibrocyte differentiation. Fibrogenesis Tissue Repair. 2010;3: 23 10.1186/1755-1536-3-23 21106092 PMC3002302

[12] Ha H, Kim KH. Pathogenesis of diabetic nephropathy: the role of oxidative stress and protein kinase C. Diabetes Res Clin Pract. 1999;45: 147–51. Available: https://www.sciencedirect.com/science/article/pii/S0168822799000443 10588367 10.1016/s0168-8227(99)00044-3

[13] Kashihara N, Haruna Y, Kondeti VK, Kanwar YS. Oxidative stress in diabetic nephropathy. Curr Med Chem. 2010;17: 4256–69. Available: http://www.ncbi.nlm.nih.gov/pubmed/20939814 20939814 10.2174/092986710793348581 PMC3708695

[14] Arnoult D, Soares F, Tattoli I, Castanier C, Philpott DJ, Girardin SE. An N-terminal addressing sequence targets NLRX1 to the mitochondrial matrix. J Cell Sci. 2009;122: 3161–8. 10.1242/jcs.051193 19692591 PMC2871076

[15] Moore CB, Bergstralh DT, Duncan JA, Lei Y, Morrison TE, Zimmermann AG, et al NLRX1 is a regulator of mitochondrial antiviral immunity. Nature. 2008;451: 573–7. 10.1038/nature06501 18200010

[16] Tattoli I, Carneiro LA, Jéhanno M, Magalhaes JG, Shu Y, Philpott DJ, et al NLRX1 is a mitochondrial NOD-like receptor that amplifies NF-kappaB and JNK pathways by inducing reactive oxygen species production. EMBO Rep. 2008;9: 293–300. 10.1038/sj.embor.7401161 18219313 PMC2267388

[17] Stokman G, Kors L, Bakker PJ, Rampanelli E, Claessen N, Teske GJD, et al NLRX1 dampens oxidative stress and apoptosis in tissue injury via control of mitochondrial activity. J Exp Med. 2017;214: 2405–2420. 10.1084/jem.20161031 28626071 PMC5551566

[18] Soares F, Tattoli I, Wortzman ME, Arnoult D, Philpott DJ, Girardin SE. NLRX1 does not inhibit MAVS-dependent antiviral signalling. Innate Immun. 2013;19: 438–448. 10.1177/1753425912467383 23212541

[19] Singh B, Saxena A. Surrogate markers of insulin resistance: A review. World J Diabetes. Baishideng Publishing Group Inc; 2010;1: 36–47. 10.4239/wjd.v1.i2.36 21537426 PMC3083884

[20] Ramakers C, Ruijter JM, Deprez RHL, Moorman AF. Assumption-free analysis of quantitative real-time polymerase chain reaction (PCR) data. Neurosci Lett. Elsevier; 2003;339: 62–66. 10.1016/S0304-3940(02)01423-4 12618301

[21] Mudaliar H, Pollock C, Panchapakesan U. Role of Toll-like receptors in diabetic nephropathy. Clin Sci (Lond). 2014;126: 685–94. 10.1042/CS20130267 24490813

[22] Brosius FC, Alpers CE, Bottinger EP, Breyer MD, Coffman TM, Gurley SB, et al Mouse Models of Diabetic Nephropathy. J Am Soc Nephrol. 2009;20: 2503–2512. 10.1681/ASN.2009070721 19729434 PMC4075053

[23] Uil M, Scantlebery AML, Butter LM, Larsen PWB, de Boer OJ, Leemans JC, et al Combining streptozotocin and unilateral nephrectomy is an effective method for inducing experimental diabetic nephropathy in the “resistant” C57Bl/6J mouse strain. Sci Rep. 2018;8: 5542 10.1038/s41598-018-23839-9 29615804 PMC5882654

[24] Ruggenenti P, Remuzzi G. Nephropathy of type 1 and type 2 diabetes: diverse pathophysiology, same treatment? Nephrol Dial Transplant. 2000;15: 1900–2. Available: http://www.ncbi.nlm.nih.gov/pubmed/11096126 11096126 10.1093/ndt/15.12.1900

[25] Kors L, Rampanelli E, Stokman G, Butter LM, Held NM, Claessen N, et al Deletion of NLRX1 increases fatty acid metabolism and prevents diet-induced hepatic steatosis and metabolic syndrome. Biochim Biophys Acta. 2018;1864: 1883–1895. 10.1016/j.bbadis.2018.03.003 29514047

[26] Costford SR, Tattoli I, Duan FT, Volchuk A, Klip A, Philpott DJ, et al Male Mice Lacking NLRX1 Are Partially Protected From High-Fat Diet–Induced Hyperglycemia. J Endocr Soc. 2018;2: 336–347. 10.1210/js.2017-00360 29577109 PMC5855099

[27] Yang XD, Tisch R, Singer SM, Cao ZA, Liblau RS, Schreiber RD, et al Effect of tumor necrosis factor alpha on insulin-dependent diabetes mellitus in NOD mice. I. The early development of autoimmunity and the diabetogenic process. J Exp Med. The Rockefeller University Press; 1994;180: 995–1004. Available: http://www.ncbi.nlm.nih.gov/pubmed/8064245 8064245 10.1084/jem.180.3.995 PMC2191653

[28] Lee L-F, Xu B, Michie SA, Beilhack GF, Warganich T, Turley S, et al The role of TNF- in the pathogenesis of type 1 diabetes in the nonobese diabetic mouse: Analysis of dendritic cell maturation. Proc Natl Acad Sci. 2005;102: 15995–16000. 10.1073/pnas.0508122102 16247001 PMC1276103

[29] Chatzantoni K, Mouzaki A. Anti-TNF-alpha antibody therapies in autoimmune diseases. Curr Top Med Chem. 2006;6: 1707–14. Available: http://www.ncbi.nlm.nih.gov/pubmed/17017952 17017952 10.2174/156802606778194217

[30] Mills EL, O’Neill LA. Reprogramming mitochondrial metabolism in macrophages as an anti-inflammatory signal. Eur J Immunol. 2016;46: 13–21. 10.1002/eji.201445427 26643360

[31] O’Neill LAJ. A Metabolic Roadblock in Inflammatory Macrophages. Cell Rep. Cell Press; 2016;17: 625–626. 10.1016/j.celrep.2016.09.085 27732839

[32] Rich PR, Rich P. The molecular machinery of Keilin’s respiratory chain An historical perspective. 2003; Available: http://www.biochemsoctrans.org/content/ppbiost/31/6/1095.full.pdf 10.1042/bst0311095 14641005

