# Supplementary figures and images for: NLRX1 does not play a role in diabetes nor the development of diabetic nephropathy induced by multiple low doses of streptozotocin
Source: PLoS One. 2019 Mar 25;14(3):e0214437. doi: 10.1371/journal.pone.0214437 (PMC6433286; doi:10.1371/journal.pone.0214437)

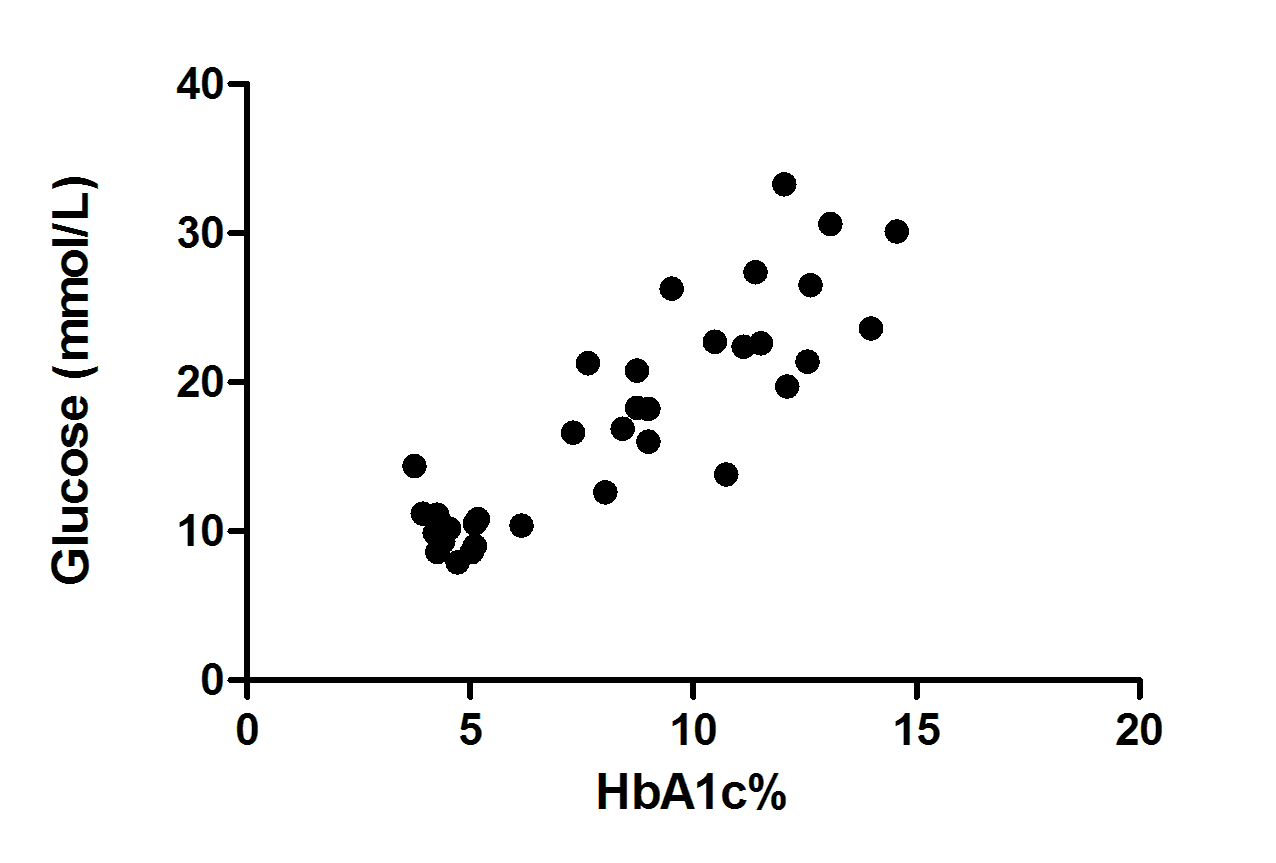

Supplement: S1 Fig — A positive correlation (R = 0.84, p<0.0001) was found between HbA1c% and fasting glucose values. (TIF) [file pone.0214437.s001.tif]
